# Electronic Cigarette Use Promotes a Unique Periodontal Microbiome

**DOI:** 10.1128/mbio.00075-22

**Published:** 2022-02-22

**Authors:** Scott C. Thomas, Fangxi Xu, Smruti Pushalkar, Ziyan Lin, Nirali Thakor, Mridula Vardhan, Zia Flaminio, Alireza Khodadadi-Jamayran, Rebeca Vasconcelos, Adenike Akapo, Erica Queiroz, Maria Bederoff, Malvin N. Janal, Yuqi Guo, Deanna Aguallo, Terry Gordon, Patricia M. Corby, Angela R. Kamer, Xin Li, Deepak Saxena

**Affiliations:** a Department of Molecular Pathobiology, New York University College of Dentistry, New York, New York, USA; b Applied Bioinformatics Labs, New York University School of Medicine, New York, New York, USA; c Department of Epidemiology & Health Promotion, New York University College of Dentistry, New York, New York, USA; d Department of Environmental Medicine, New York University School of Medicine, New York, New York, USA; e Department of Oral Medicine, School of Dental Medicine, University of Pennsylvania, Philadelphia, Pennsylvania, USA; f Department of Periodontology and Implant Dentistry, New York University College of Dentistry, New York, New York, USA; Vallabhbhai Patel Chest Institute

**Keywords:** dysbiosis, electronic cigarette, microbiome, periodontitis, subgingival plaque

## Abstract

Electronic cigarettes (e-cigs) have become prevalent as an alternative to conventional cigarette smoking, particularly in youth. E-cig aerosols contain unique chemicals which alter the oral microbiome and promote dysbiosis in ways we are just beginning to investigate. We conducted a 6-month longitudinal study involving 84 subjects who were either e-cig users, conventional smokers, or nonsmokers. Periodontal condition, cytokine levels, and subgingival microbial community composition were assessed, with periodontal, clinical, and cytokine measures reflecting cohort habit and positively correlating with pathogenic taxa (e.g., Treponema, *Saccharibacteria*, and *Porphyromonas*). α-Diversity increased similarly across cohorts longitudinally, yet each cohort maintained a unique microbiome. The e-cig microbiome shared many characteristics with the microbiome of conventional smokers and some with nonsmokers, yet it maintained a unique subgingival microbial community enriched in *Fusobacterium* and *Bacteroidales* (G-2). Our data suggest that e-cig use promotes a unique periodontal microbiome, existing as a stable heterogeneous state between those of conventional smokers and nonsmokers and presenting unique oral health challenges.

## INTRODUCTION

The use of electronic cigarettes (e-cigs) is on the rise in adults and young people (1–7). The use of e-cigs is often perceived and promoted as a safer alternative to cigarette smoking due to the absence or reduction of harmful combustion products (4, 8–10). Yet e-cigs produce some toxic compounds shared with cigarettes in addition to unique products (11–16). E-cigs work by aerosolizing a liquid that contains nicotine, propylene glycol, and glycerol (flavoring chemicals are common) that is then inhaled by the user (17, 18), whereby the mouth and oral microbial community are the first exposed. As personal perception of the safety of e-cigs is a major determiner of use (4, 19, 20), continued research on the impact of e-cig use on oral and human health is warranted (17, 21).

The periodontium components include gingival tissues and alveolar bone, which support the teeth and create a unique subgingival physicochemical habitat that harbors a diverse microbial community in direct contact with tissue and tooth, the diversity of which can be diagnostic of oral health (22, 23). This habitat is impacted by the myriad molecules entering the human oral cavity (24, 25). Periodontitis arises from imbalances of the microbial community (dysbiosis) inhabiting the periodontal pocket and subsequent host immune and inflammatory responses, leading to the destruction of tissues and necessary medical intervention to avoid tooth loss and systemic disease (25–30).

Conventional cigarette smoking is a risk factor for periodontitis, causing detrimental changes in the composition of the oral microbiome, promoting an inflammatory response, inhibiting the immune system, and promoting bone loss (31–34). Unlike for conventional cigarette use, the effects of e-cig use on subgingival microbial community composition and diseases are underinvestigated (5, 6, 35–37). Previously we have shown in a cross-sectional study using saliva samples that e-cig aerosol can alter the salivary microbiome and host inflammatory response (6). Epithelial cells exposed to e-cig aerosols are more prone to infection, with *in vitro* infection models using oral pathogens in the presence of e-cig aerosols stimulating the inflammatory response (6). Other studies have also suggested that e-cig aerosols can alter the balance of the oral microbial community, with effects on epigingival tissue (18, 38, 39). Yet there is no clear consensus on what a dysbiotic, chronically e-cig-exposed, periodontal microbial community looks like and if it bears similarities to that of at-risk conventional smokers.

Here, we report on a longitudinal clinical study evaluating the adverse effects of e-cig use on periodontal health. We investigated the effects of e-cig use on the composition of the human subgingival plaque (SGP) microbial community of 84 subjects over a 6-month interval, integrating microbiome data with clinical measures and SGP cytokine concentrations. All subjects presented with at least mild periodontitis and did not receive prophylactic cleaning during the study period, providing an opportunity to compare alterations of a dysbiotic subgingival microbial community due to habit and monitor disease progression. We compared conventional cigarette smokers (CS; *n* = 27), e-cig-only users (ES; *n* = 28), and nonsmokers (NS; *n* = 29) to assess the degree to which the e-cig subgingival microbiome resembles those of conventional smokers and nonsmokers. Our data suggest that e-cig use promotes a stable periodontal microbiome that is between those of the conventional cigarette smoker and nonsmoker and has unique features that may impact host oral health in a manner different than conventional cigarette use.

## RESULTS

### Clinical data demonstrate periodontitis severity and delineate cohorts.

**FIG 1:**
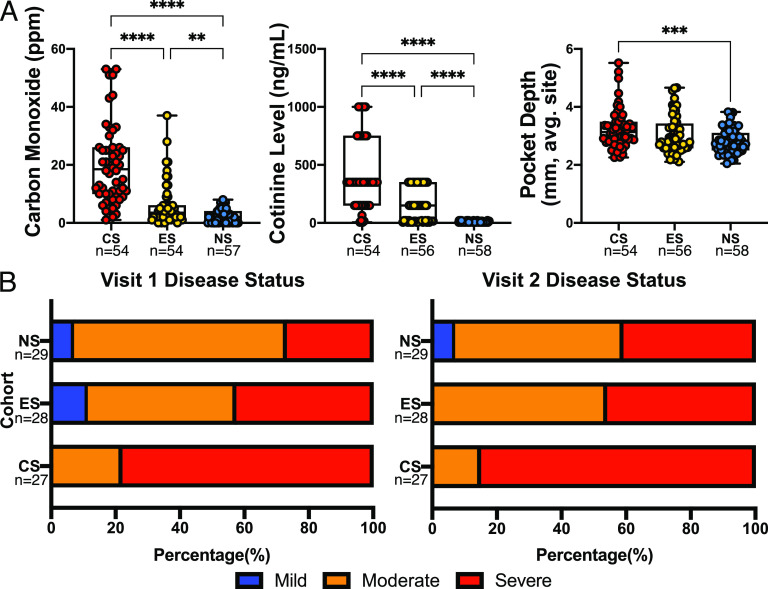
Clinical measures validate patient inclusion and demonstrate disease status and progression in specific cohorts. (A) Patient breath carbon monoxide levels in parts per million, saliva cotinine concentration, and the average distance from the free gingival margin to the depth of the pocket (pocket depth); sample number is given below cohort designation. Kruskal-Wallis H with *post hoc* Dunn’s test was performed, with multiplicity-adjusted *P* values reported. **, *P* < 0.01; ***, *P* < 0.001; ****, *P* < 0.0001. On each visit, patients were evaluated for the degree of periodontitis, as described by Xu et al. (7) (B). CS, conventional cigarette smokers; ES, e-cigarette users; NS, nonsmokers. Number of subjects is given below each cohort.

As expected, patient breath carbon monoxide and saliva cotinine levels were significantly lower in the NS than in the ES or CS cohort, with those for the CS also significantly higher than for ES (Fig. 1A). Pocket depth, a measure of disease severity (7), was significantly higher in the CS than in the NS. Periodontal conditions were classified into distinct categories based on clinical measures (Fig. 1B; see also Text S1 in the supplemental material) (7). The CS cohort predominately consisted of subjects with severe periodontitis for both visits, containing no patients with mild periodontitis at either visit. The ES cohort had a higher percentage of severe periodontitis at both visits than did the NS cohort. Despite not smoking, the NS cohort consisted predominately of patients with moderate periodontitis at both visits. The ES cohort had three subjects progress from mild to moderate periodontitis, and one subject progressed from moderate to severe. Four patients each from the CS and the NS progressed from moderate to severe periodontitis. Table 1 shows patient demographics for each cohort.

**TABLE 1:** Patient demographics

Characteristic	Value for:		
	Cigarette smokers	E-cigarette users	Nonsmokers
No.	27	28	29
Sex (% male)	81.48	78.57	55.17
Age (yrs), mean (SD)			
Female	51 (10.3)	39.7 (11.3)	38.9 (14.9)
Male	48.2 (9.51)	35.8 (10.3)	28.6 (7.05)
Ethnicity (% Hispanic)	7.41	17.86	24.14
Race (%)			
White	33.33	57.14	27.59
Black	59.26	35.71	31.03
Asian	3.70	7.14	37.93
Other	3.70	0.00	3.45

10.1128/mbio.00075-22.1 TEXT S1 Study eligibility, classification criteria, and study procedures. Download Text S1, DOCX file, 0.02 MB. Copyright © 2022 Thomas et al. 2022 Thomas et al. https://creativecommons.org/licenses/by/4.0/ This content is distributed under the terms of the Creative Commons Attribution 4.0 International license.

### E-cig users harbor a unique subgingival microbiome.

**FIG 2:**
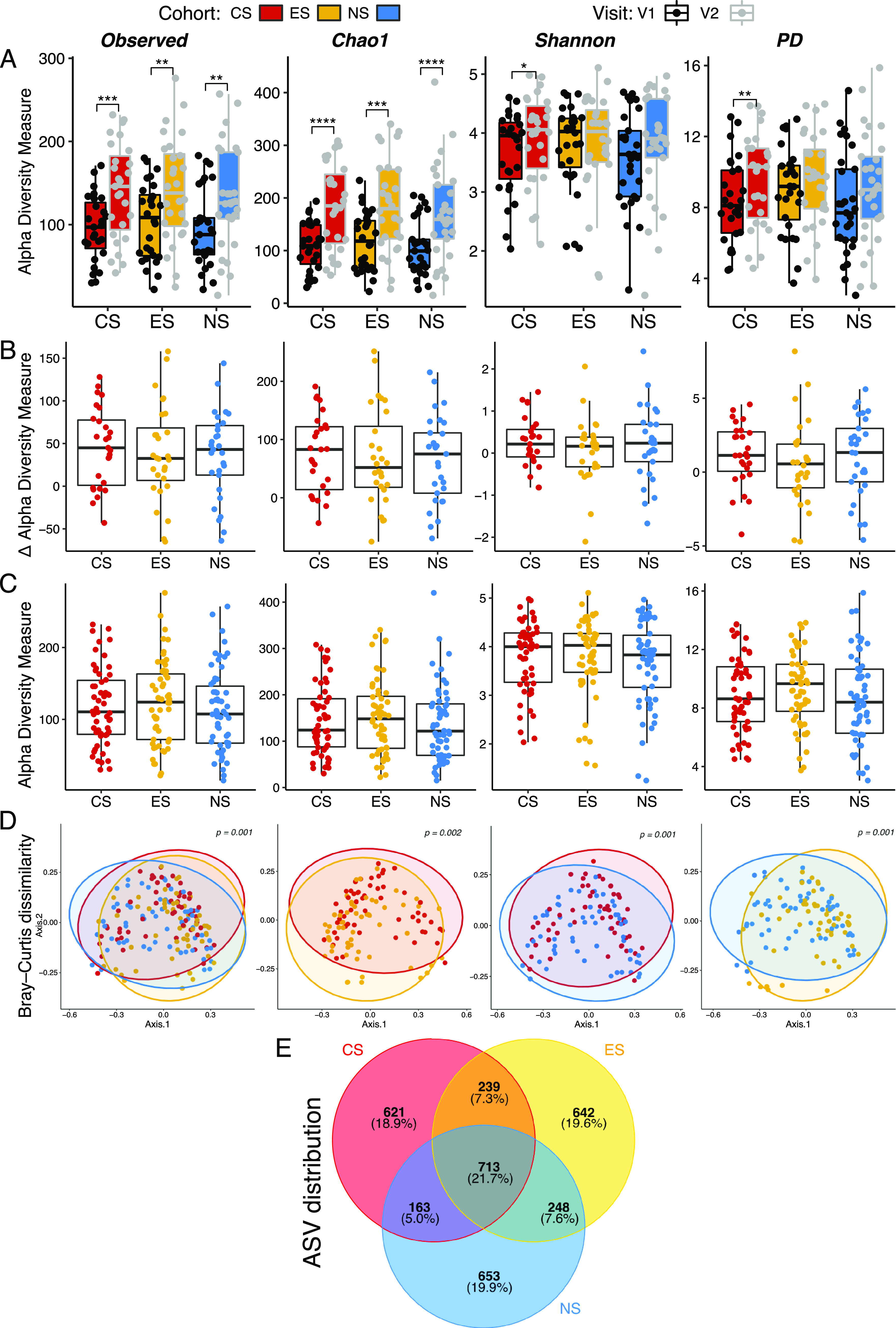
Periodontal microbiome α-diversity remains similar among cohorts, yet community structure is unique. α- and β-diversities of periodontal microbial communities for conventional cigarette smokers, e-cigarette users, and nonsmokers are shown. Sample numbers are provided in Materials and Methods. α-Diversity between visits tended to increase within a cohort (A) (paired-sample Wilcoxon). The degree of change in α-diversity measures between visits (v2 – v1) for a given cohort was not significantly different between the cohorts (B) (Mann-Whitney U test). When visits were merged within a cohort, no significant differences in α-diversity were observed (C). Mean and SEM are shown for panels A to C; box indicates the interquartile range of the data. β-Diversity of periodontal microbial communities was significantly different between cohorts (PERMANOVA) (D). *, *P* < 0.05; **, *P* < 0.01; ***, *P* < 0.001; ****, *P* < 0.0001. A Venn diagram depicts shared and unique amplicon sequence variants (ASVs) among the cohorts; percentages of total ASVs are in parentheses (E).

We evaluated changes in α-diversity both within and across cohorts. Observed and predicted richnesses significantly increased in all cohorts between visit 1 (v1) and visit 2 (v2) (Fig. 2A), while Shannon and Faith's phylogenetic diversity measures significantly increased only in the CS. When evaluating α-diversity measures across cohorts for either v1 or v2, no significant differences were observed between cohorts (Fig. S1). We then observed the magnitude of change between v2 and v1 based on per-patient differences (v2 − v1), with no significant differences in the change of α-diversity measure observed between any of the cohorts (Fig. 2B) (Wilcoxon rank-sum). Therefore, we combined visits for further analysis to increase our sample size and statistical power (40). When both visits were accounted for within a cohort, no significant differences were found in α-diversity across cohorts (Fig. 2C), indicating that similar numbers of taxa were found in the cohorts.

10.1128/mbio.00075-22.2 FIG S1 α-Diversity measures for visit 1 (A) or visit 2 (B) did not significantly differ between cohorts consisting of conventional smokers (CS; *n* = 27), e-cig users (ES; *n* = 28), and nonsmokers (NS; *n* = 29). Download FIG S1, EPS file, 1.1 MB. Copyright © 2022 Thomas et al. 2022 Thomas et al. https://creativecommons.org/licenses/by/4.0/ This content is distributed under the terms of the Creative Commons Attribution 4.0 International license.

To compare community structure between cohorts and visits, we examined differences in β-diversity. When visits were grouped within a cohort, significant differences were found between cohorts (Fig. 2D). All cohorts exhibited distinct microbial communities from one another, as demonstrated by significance in all possible pairwise comparisons (Fig. 2D). No significant differences were found between visits for any cohort (Fig. S2), further justifying grouping of visits within a cohort. All cohorts were found to be significantly different when evaluating β-diversity across cohorts on a per-visit basis, except when comparing v2 between the ES and CS (*P* = 0.065 [Fig. S2]). These results demonstrate that microbiome structure did not significantly differ within a cohort, despite changes in α-diversity. Additionally, each cohort was found to host a unique microbiome, whether visits were grouped within a cohort or evaluated on a per-visit basis, except for the ES and the CS after prolonged cigarette use (v2).

10.1128/mbio.00075-22.3 FIG S2 β-Diversity between visits did not significantly differ within a cohort but did across cohorts. Shown are Bray-Curtis PCoA comparing visit 1 and visit 2 within a cohort and across cohorts. Within a cohort, no significant differences were observed between visits (A). For visit 1, all cohorts significantly differed from one another (B). For visit 2, the CS and NS cohorts and ES and NS cohorts significantly differed (C). Download FIG S2, TIF file, 0.9 MB. Copyright © 2022 Thomas et al. 2022 Thomas et al. https://creativecommons.org/licenses/by/4.0/ This content is distributed under the terms of the Creative Commons Attribution 4.0 International license.

A total of 3,279 amplicon sequence variants (ASVs) were observed across all cohorts, with ∼19.4% (±0.5%) of them unique to a particular cohort (Fig. 2E). Core ASVs found in all three cohorts accounted for 21.7% of total ASVs. Notably, while the percentages of ASVs shared between the CS and ES or the NS and ES were similar (7.3 and 7.6%, respectively), the percentage shared between the CS and NS was lower (5.0%). These results demonstrate that while there is a core periodontal microbiome between cohorts, each cohort has unique features, with the CS sharing more in common with the ES than the NS.

### Patterns in relative abundance expose uniqueness and commonalities in e-cig microbiomes.

**FIG 3:**
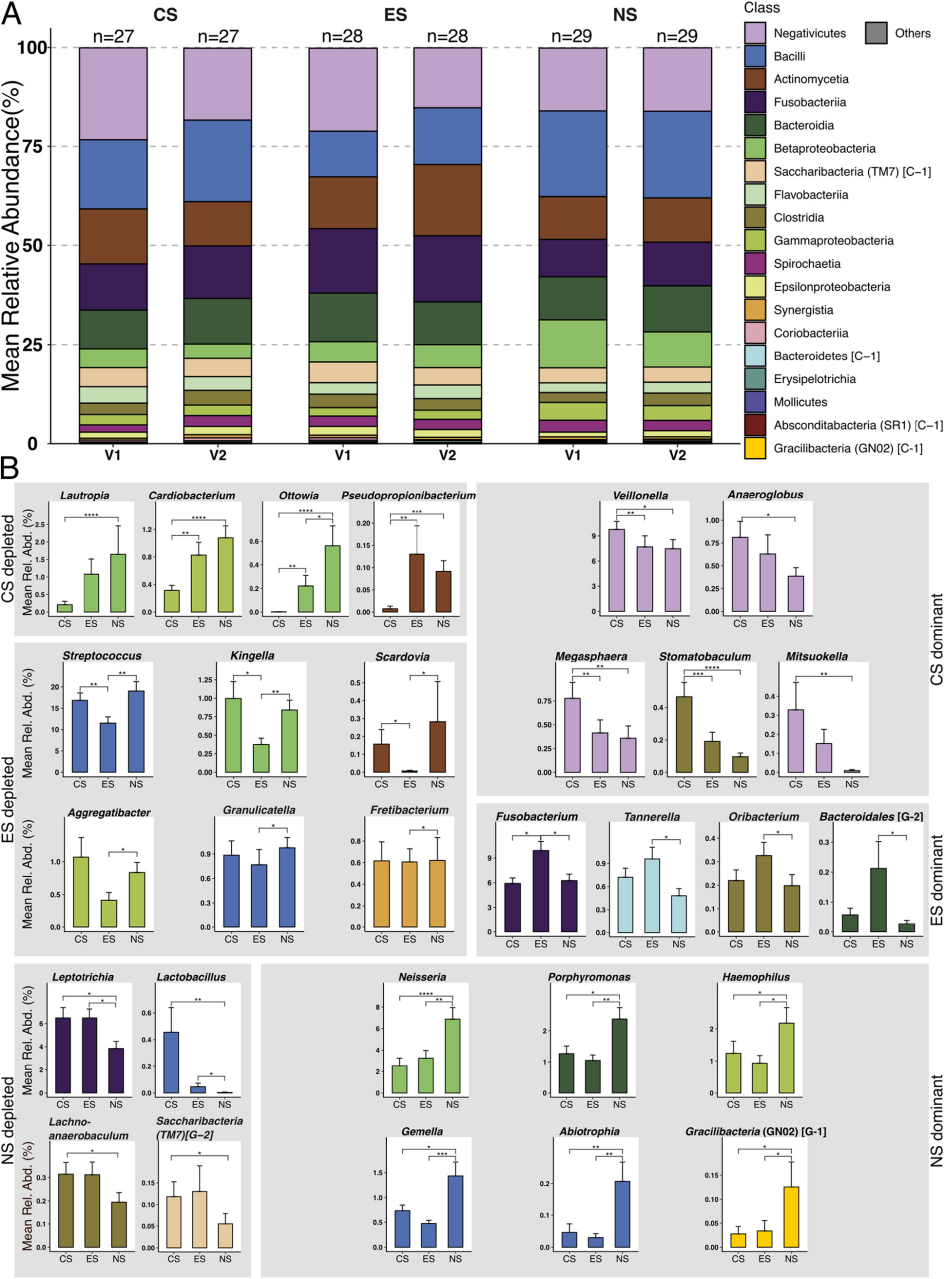
Cohorts displayed distinct patterns in taxa mean relative abundance. Periodontal microbial community composition at the class level and genera-based differential relative abundance are shown for CS, ES, and NS. (A) Per-visit bar plots for relatively abundant classes in the different cohorts. (B) Statistically significant differentially relatively abundant genera grouped based on abundance patterns in the three cohorts (visits merged within a cohort) (Mann-Whitney U test; *, *P* < 0.05; **, *P* < 0.01; ***, *P* < 0.001). Rel. Abd., relative abundance.

There were 19 classes above 0.1% (mean) relative abundance in at least one cohort and visit (Fig. 3A). The *Negativicutes*, *Bacilli*, *Actinomycetia* (41), *Fusobacteria*, *Bacteroidia*, and *Betaproteobacteria* all had greater than 10% relative abundance in at least one cohort and visit and, taken together, accounted for greater than 78% of relative abundance in any cohort and visit. The classes *Synergistia*, *Coriobacteriia*, *Bacteroidetes* (C-1), *Erysipelotrichia*, *Absconditabacteria* (SR1, C-1), *Mollicutes*, and *Gracilibacteria* (GN02, C-1) all had less than 1.0% relative abundance across all cohorts and visits.

Within these 19 classes, 59 genera were above 0.1% relative abundance in at least one cohort. Streptococcus, *Veillonella*, *Fusobacterium*, *Prevotella*, *Rothia*, *Selenomonas*, *Leptotrichia*, and *Neisseria* all had greater than 5% relative abundance in at least one of the cohorts, with the genus Streptococcus having greater than 10% in all cohorts.

Genera abundance patterns fell into seven general categories when cohort visits were merged and analyzed for significant differences in relative abundance between cohorts. A genus was either significantly enriched or depleted in a given cohort (Fig. 3B), or no significant difference was found (genera not shown). A total of 29 genera were significantly differentially abundant between cohorts (Fig. 3B). All significant differences were additionally supported by linear discriminant analysis effect size (LEfSe) analysis.

The genera *Lautropia*, *Cardiobacterium*, *Ottowia*, and *Pseudopropionibacterium* were significantly depleted in the CS compared to the ES and NS and, apart from *Pseudopropionibacterium*, showed a pattern of enrichment from the ES to the NS (Fig. 3B). *Lautropia* consisted of 10 ASVs, all of which were classified as Lautropia mirabilis. *Cardiobacterium* contained 45 ASVs, with only ASVs classified as Cardiobacterium hominis or C. valvarum significantly different between cohorts. *Ottowia* consisted of 11 ASVs, all classified as unclassified human microbial taxon (HMT) 894. *Pseudopropionibacterium* contained 7 ASVs, classified as P. propionicum and unclassified species HMT 194.

In contrast, *Veillonella*, *Megasphaera*, and *Stomatobaculum* were significantly enriched in the CS compared to ES and NS, with *Anaeroglobus* and *Mitsuokella* also being significantly enriched compared to the NS. *Veillonella* consisted of 99 ASVs, with 12 ASVs belonging to Veillonella parvula, V. atypica, V. dispar, or unclassified being significantly different between cohorts. *Megasphaera* consisted of 23 ASVs, predominately composed of Megasphaera micronuciformis and an unclassified species HMT 123. *Stomatobaculum* consisted of 13 ASVs, consisting predominately of Stomatobaculum longum and an unclassified species HMT 910. *Anaeroglobus* consisted of 14 ASVs, all of which were classified as A. geminatus. *Mitsuokella* consisted of 11 ASVs composed of unclassified species HMT 131 and HMT 521.

The genera Streptococcus, *Kingella*, and *Scardovia* were significantly depleted in the ES compared to CS or NS, with *Aggregatibacter*, *Granulicatella*, and *Fretibacterium* also being significantly depleted compared to NS. Streptococcus consisted of 73 ASVs, with 8 ASVs belonging to Streptococcus parasanguinis (clade 411), S. sanguinis, S. gordonii, S. anginosus, and unclassified species being significantly different between cohorts. *Kingella* consisted of 17 ASVs, predominantly composed of Kingella denitrificans, K. oralis, and an unclassified species HMT 012. *Scardovia* consisted of 3 ASVs belonging to Scardovia wiggsiae and S. inopinata. *Aggregatibacter* consisted of 44 ASVs, with a single unclassified ASV significantly enriched in the NS than ES. *Granulicatella* consisted of 11 ASVs classified as G. adiacens or G. elegans. *Fretibacterium* consisted of 19 ASVs classified as Fretibacterium fastidiosum as well as unclassified species (including HMT 362 and HMT 361).

In contrast, *Fusobacterium* was significantly enriched in ES compared to CS and NS. *Tannerella*, *Oribacterium*, and *Bacteroidales* (G-2) were significantly enriched in ES with respect to NS. *Fusobacterium* consisted of 182 ASVs, with 13 ASVs belonging to Fusobacterium nucleatum (Fusobacterium nucleatum subsp. *vincentii* and Fusobacterium nucleatum subsp. *animalis*) and unclassified species (including HMT 203) being significantly different between cohorts. *Tannerella* consisted of 44 ASVs consisting of Tannerella forsythia and unclassified species (including HMT 286, HMT 808, and HMT 916). *Oribacterium* consisted of 9 ASVs, composed of Oribacterium asaccharolyticum and unclassified species (including HMT 078 and HMT 102). *Bacteroidales* (G-2) consisted of 7 ASVs, all belonging to an unclassified species HMT 274.

The genera *Leptotrichia* and *Lactobacillus* were significantly depleted in NS compared to CS and ES, with *Lachnoanaerobaculum* and *Saccharibacteria* (TM7) (G-2) significantly depleted compared to CS. *Leptotrichia* consisted of 182 ASVs, with 13 ASVs belonging to L. hongkongensis, L. goodfellowii, L. wadei, L. shahii, and unclassified species (HMT 225, HMT 392, and HMT 212) significantly different between cohorts. *Lactobacillus* consisted of 18 ASVs, with an ASV for L. gasseri and an unclassified ASV significantly different between cohorts. *Lachnoanaerobaculum* consisted of 39 ASVs classified as Lachnoanaerobaculum orale, L. saburreum, L. umeaense, or unclassified species (including HMT 083, HMT 089, and HMT 496). *Saccharibacteria* (TM7) (G-2) consisted of a single ASV identified as unclassified HMT 350.

In contrast, the *Neisseria*, *Porphyromonas*, Haemophilus, *Gemella*, *Abiotrophia*, and *Gracilibacteria* (GN02) (G-1) were significantly enriched in NS compared to CS and ES, with CS and ES displaying similar relative abundances. *Neisseria* consisted of 72 ASVs, with 13 ASVs belonging to Neisseria bacilliformis, N. oralis, N. elongata, N. subflava, and unclassified species significantly different between cohorts. *Porphyromonas* consisted of 87 ASVs, with 3 ASVs belonging to Porphyromonas catoniae, P. pasteri, and unclassified species HMT 278 significantly different between cohorts. Haemophilus consisted of 29 ASVs, with 3 ASVs belonging to Haemophilus parainfluenzae and an unclassified species significantly different between cohorts. *Gemella* consisted of 16 ASVs predominated by Gemella morbillorum, with 2 ASV belonging to G. morbillorum and an unclassified species significantly different between cohorts. *Abiotrophia* consisted of 4 ASVs, all belonging to Abiotrophia defectiva. *Gracilibacteria* (GN02) (G-1) consisted of 7 ASVs, with 6 belonging to HMT 872 and 1 to HMT 871.

**FIG 4:**
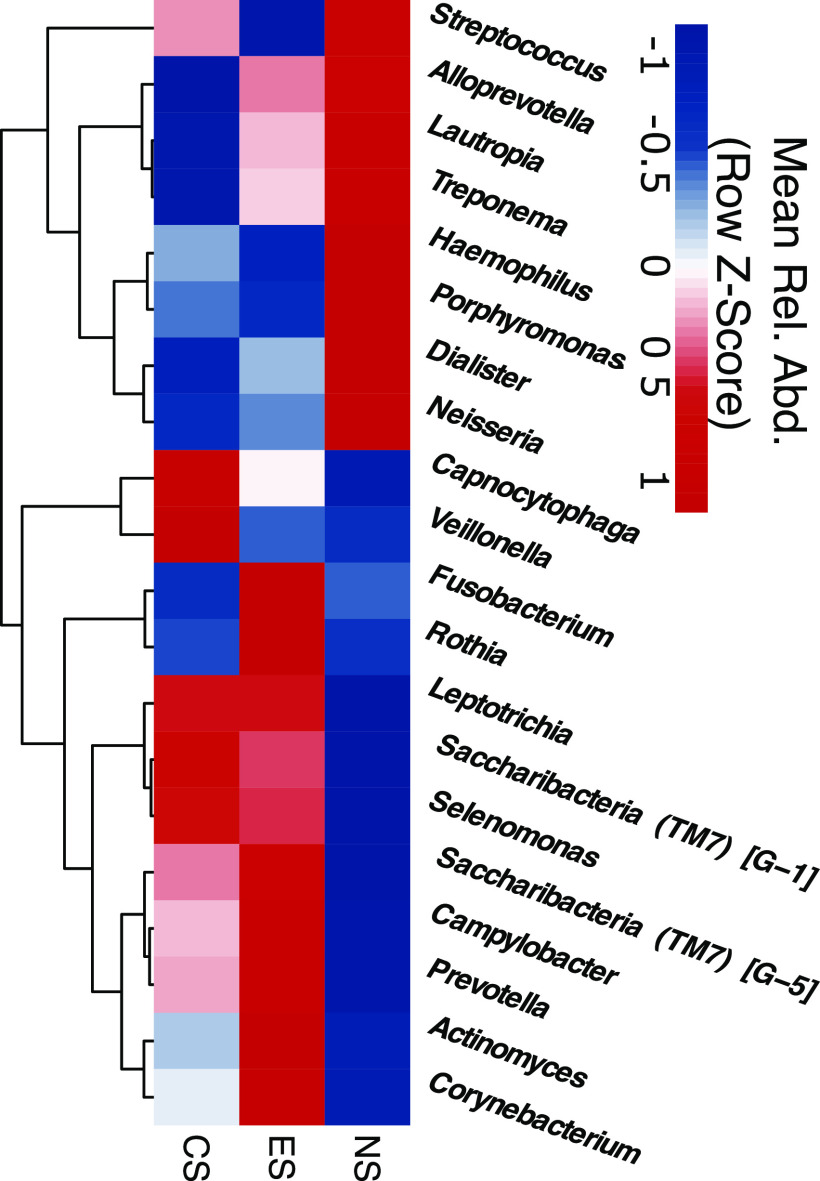
Mean-relative-abundance patterns for genera distinguish nonsmokers from e-cig users and conventional smokers. A hierarchical-clustering relative-abundance heat map of the 20 most relatively abundant genera in the three cohorts with row z-score is displayed.

Hierarchical clustering of the 20 most relatively abundant genera displayed clear partitioning of taxa based on their relative abundance in NS or ES and CS (Fig. 4). The genera Streptococcus, *Alloprevotella*, *Lautropia*, Treponema, Haemophilus, *Porphyromonas*, *Dialister*, and *Neisseria* clustered together with a higher-than-mean relative abundance in NS than in CS and ES. In contrast, *Fusobacterium*, *Rothia*, *Leptotrichia*, *Saccharibacteria* (TM7) (G-1 and G-5), *Selenomonas*, Campylobacter, *Prevotella*, *Actinomyces*, and *Corynebacterium* all clustered together with a higher-than-mean relative abundance in ES than in NS, with mixed-abundance patterns for these genera in CS. The genera *Capnocytophaga* and *Veillonella* formed a high-level cluster with a higher mean relative abundance in CS than in ES or NS.

### E-cig users share similar ASV relative-abundance patterns with conventional smokers.

**FIG 5:**
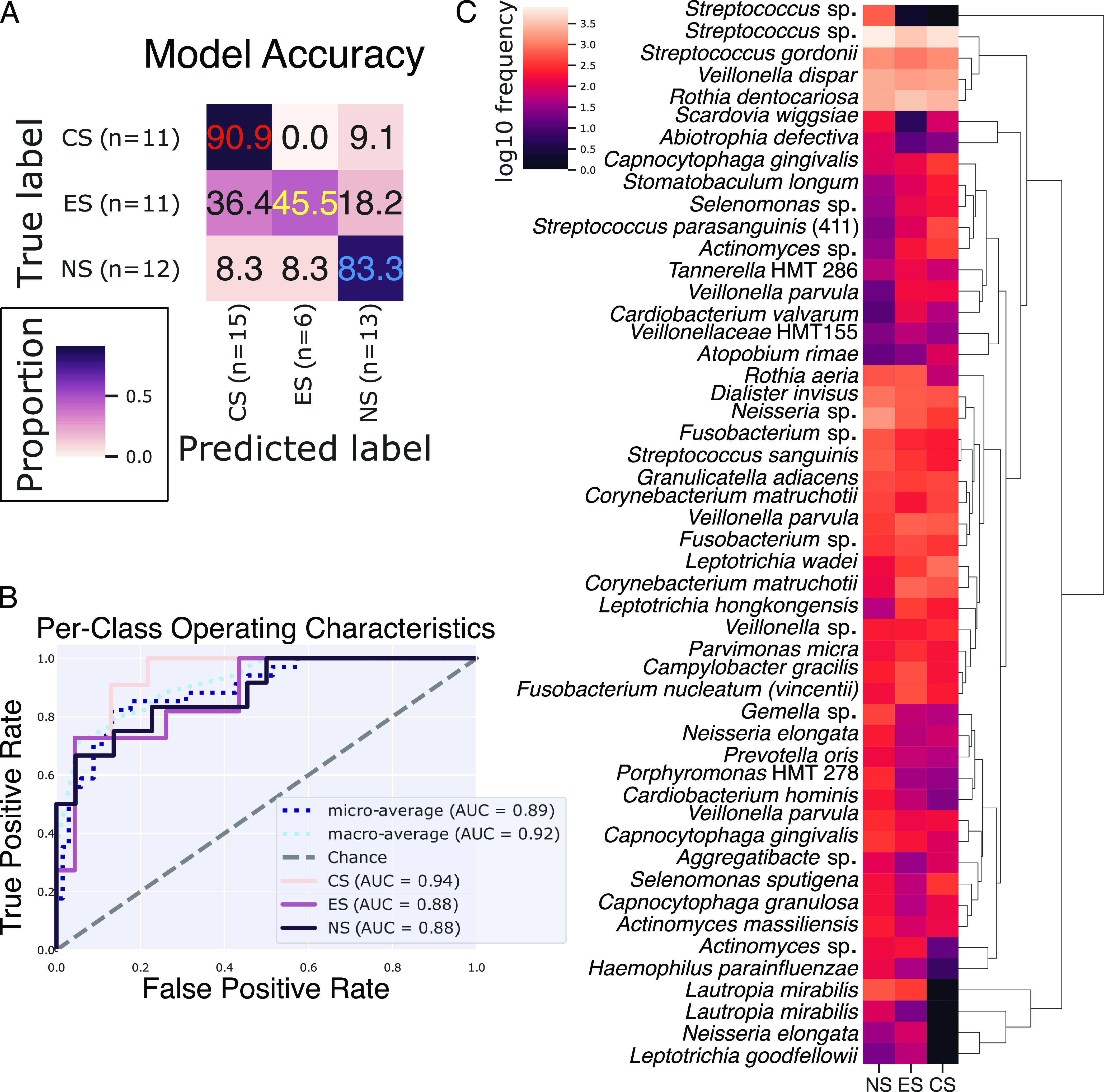
The e-cig user periodontal microbiome resembles those of both conventional smokers and nonsmokers. Supervised learning sample classification can accurately predict sample inclusion in the CS and NS but struggles with the ES cohort (A). The model accuracy was tested on 34 samples that were excluded from the training data set (*n* = 134). All cohorts had areas under the curve (AUC) well above what would be expected by chance (B). A hierarchical-clustering mean-relative-abundance heat map of the 50 most important features (ASVs) shows important clusters with distinct abundance patterns and pathological relevance (C).

We used supervised machine learning to evaluate the uniqueness of cohort microbiomes (Fig. 5). The resulting model was the most successful at predicting inclusion in the CS cohort (90.9% accuracy), with findings for the NS cohort being slightly less accurate (83.3%) (Fig. 5A). In contrast, the ability of the model to correctly predict inclusion in the ES cohort was relatively low (45.5%), incorrectly assigning samples to the CS and NS cohorts 36.4% and 18.2% of the time (respectively), resulting in an overall model accuracy of 73.5%. The per-class areas under the curve for receiver operating characteristic graphs were 0.94, 0.88, and 0.88 for CS, ES, and NS, respectively (Fig. 5B).

Hierarchical clustering of the top 50 ASVs for predicting sample inclusion in a cohort contained several known pathogens and commensals, including clusters with highly enriched and depleted taxa (Fig. 5C). Lautropia mirabilis, Neisseria elongata, and Leptotrichia goodfellowii formed a high-level cluster found at relatively moderate abundance in NS and ES yet nearly absent in CS. An unclassified Streptococcus sp. clustered alone and was depleted in the CS and ES cohorts yet enriched in NS. Another high-level cluster contained enriched ASVs classified as an unclassified Streptococcus sp., S. gordonii, Veillonella dispar, and Rothia dentocariosa. Parvimonas micra, Campylobacter gracilis, and F. nucleatum subsp. *vincentii* formed a lower-level cluster where relative abundance in ES was greater than in NS or CS. Leptotrichia wadei, Corynebacterium matruchotii, and Leptotrichia hongkongensis also formed a lower-level cluster where NS tended to be depleted compared to the case with cigarette user cohorts. Both commensals and pathogens and low- and high-relative-abundance organisms contribute to the uniqueness of a cohort’s microbiome, yet accurately classifying e-cig users was problematic, with them often being classified as conventional smokers.

### Genera correlate with cytokine and clinical measures.

**FIG 6:**
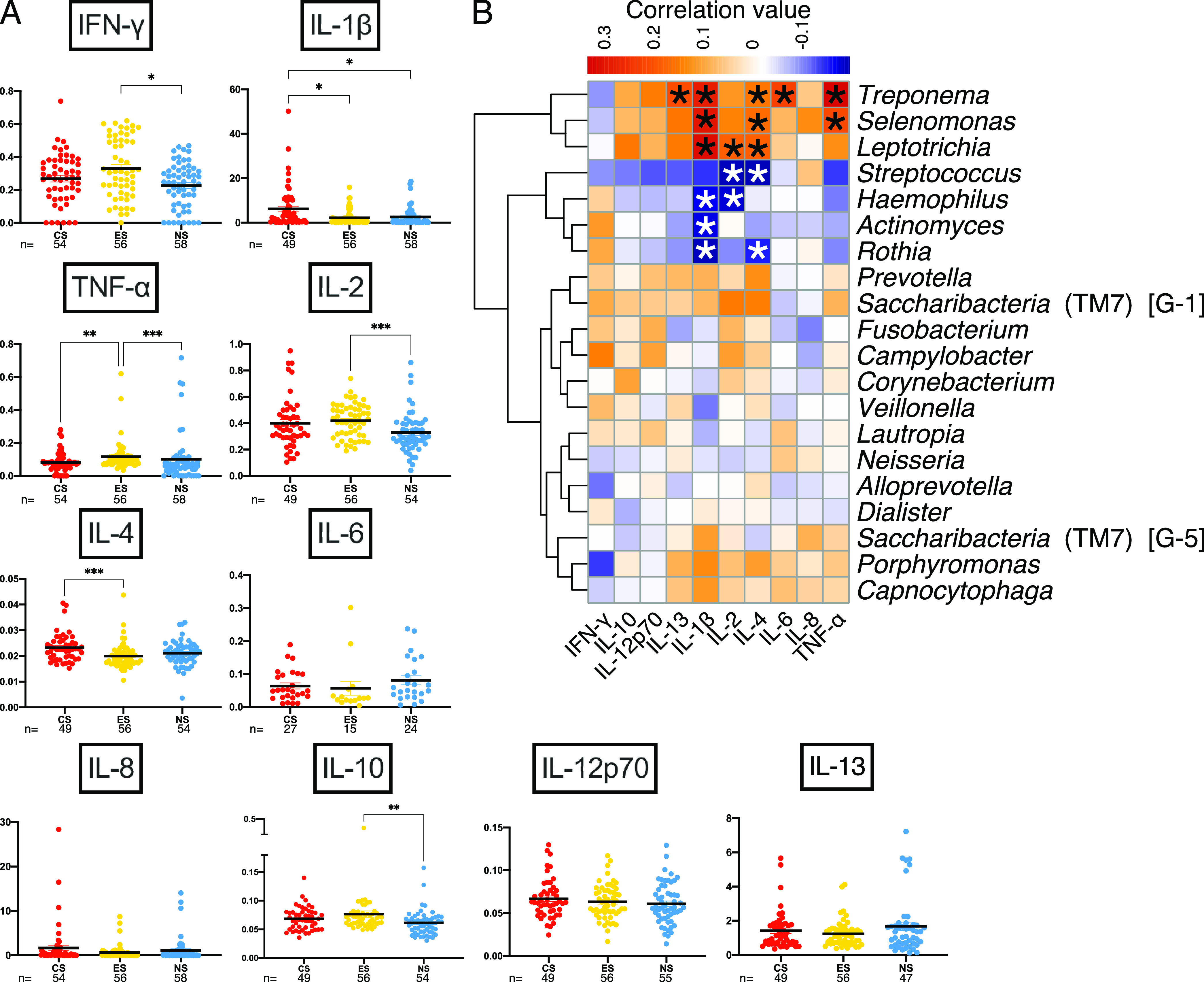
Cytokine abundance patterns differ among cohorts, and cytokines positively correlate with known pathogens. (A) Cytokine concentrations (picograms per milliliter) for the three cohorts; sample numbers are displayed below cohort designation. Kruskal-Wallis H with *post hoc* Dunn’s test was performed, with multiplicity-adjusted *P* values reported. *, *P* < 0.05; **, *P* < 0.01; ***, *P* < 0.001. (B) Hierarchical-clustering correlation heat map with cytokines and the 20 most relatively abundant genera. *, *P* < 0.05.

We performed a Pearson correlation analysis to evaluate how cytokines and clinical measures correlated with prominent genera. Multiple cytokines were found to significantly differ in concentration among the three cohorts (Fig. 6A). Interleukin 1β (IL-1β) was significantly higher in CS than in ES or NS. Gamma interferon (IFN-γ), IL-2, and IL-10 were significantly higher in ES than NS, with CS having a higher average than NS. Tumor necrosis factor alpha (TNF-α) was significantly higher in ES than CS or NS. IL-4 was significantly higher in CS than ES, where CS had a higher average than NS. There were no significant differences in cytokine concentrations between cohorts for IL-6, IL-8, IL-12p70, or IL-13.

Hierarchical clustering of correlation analyses between proinflammatory cytokines and the top 20 most abundant genera indicated a cluster of three genera with relatively high positive correlations between Treponema, *Selenomonas*, and *Leptotrichia* and all cytokines except IFN-γ and, in the case of *Leptotrichia*, IL-8 (Fig. 6B). These three genera showed robust positive correlations with IL-1β and, to a slightly lower degree, TNF-α. *Porphyromonas* and *Capnocytophaga* clustered together with positive correlations with IL-13, IL-1β, IL-2, IL-4, IL-6, IL-8, and TNF-α. *Prevotella* and *Saccharibacteria* (TM7) (G-1) formed a cluster that was positively correlated with IFN-γ, IL-10, IL-12p70, IL-13, IL-1β, IL-2, IL-4, and TNF-α. Streptococcus, Haemophilus, *Actinomyces*, and *Rothia* formed a cluster that was noticeably negatively correlated with IL-1β.

**FIG 7:**
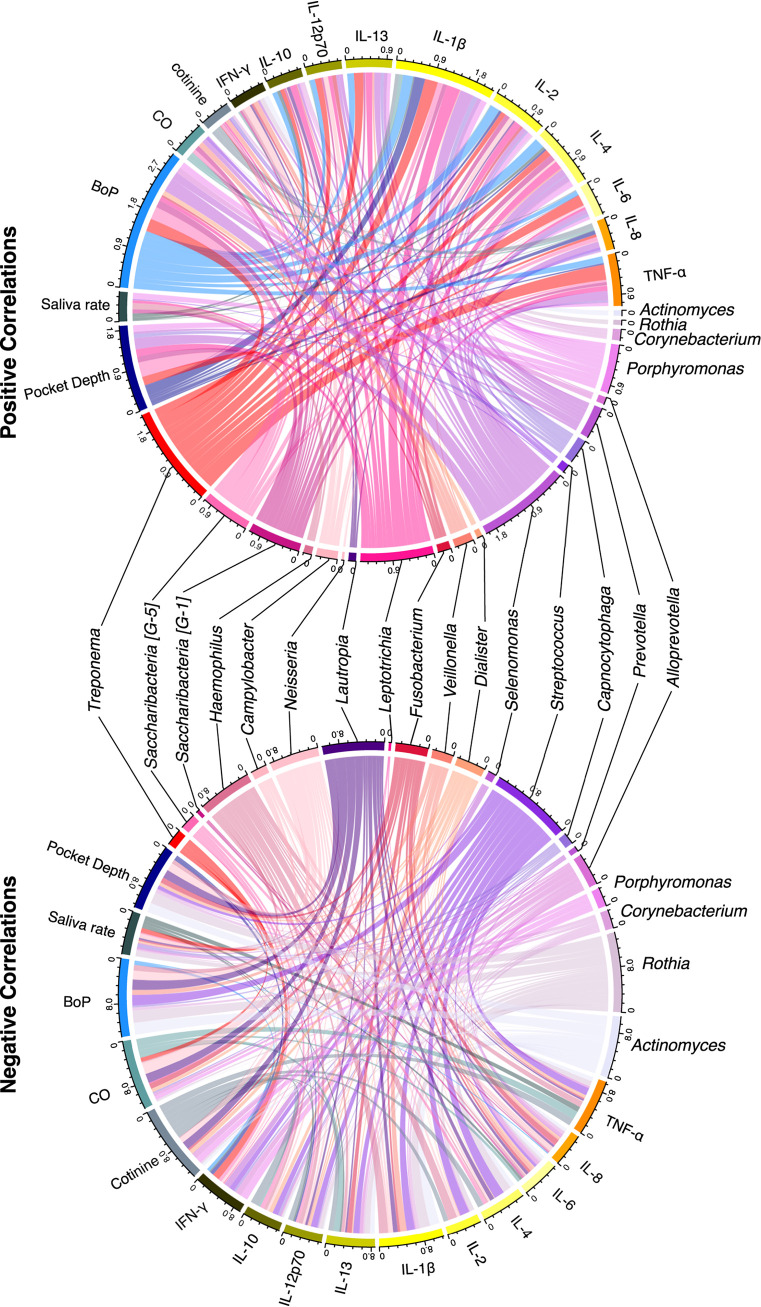
Pathogens and commensals correlate with clinical measures and cytokines. Positive and negative correlation cord diagrams demonstrate correlations between matched samples for the 20 most abundant genera, clinical measures of periodontal disease, and cytokines. BoP, bleeding on probing; CO, carbon monoxide.

Correlations between the top 20 most relatively abundant genera, cytokine concentrations, and clinical measures showed patterns of pathogenic genera being positively correlated with cytokines and clinical measures (Fig. 7 and Table S1). The genera Treponema, *Selenomonas*, *Leptotrichia*, *Porphyromonas*, and *Saccharibacteria* (G-5 and G-1) had numerous and fair positive correlations between clinical and cytokine measures (42). Treponema positively correlated primarily with TNF-α, IL-1β, and IL-6 and with all other cytokines except IFN-γ. Additionally, Treponema significantly correlated with the clinical measures of bleeding on probing rate and pocket depth, both of which are strong indicators of periodontitis. *Selenomonas* correlated with numerous measures, especially with pocket depth, bleeding on probing, and IL-1β. *Leptotrichia* correlated with IL-1 and, to a lesser degree, numerous other measures. *Porphyromonas* correlated with pocket depth, saliva rate, and bleeding on probing, as well as numerous cytokines to a lesser degree. *Saccharibacteria* G-5 significantly correlated with bleeding on probing and pocket depth, while G-1 correlated to a lesser degree with more measures.

10.1128/mbio.00075-22.4 TABLE S1 Correlation coefficients and associated significance for clinical measures, cytokine concentration, and microbiota. Download Table S1, XLSX file, 0.02 MB. Copyright © 2022 Thomas et al. 2022 Thomas et al. https://creativecommons.org/licenses/by/4.0/ This content is distributed under the terms of the Creative Commons Attribution 4.0 International license.

Some commensal genera showed negative correlations (Fig. 7) with cytokines and clinical measures. The genera *Actinomyces*, *Rothia*, Streptococcus, and *Lautropia* had numerous and fair negative correlations. *Actinomyces* and *Rothia* significantly negatively correlated with the clinical measures of bleeding on probing rate and pocket depth and the cytokine IL-1β and to a lesser degree with numerous other measures. Streptococcus primarily negatively correlated with bleeding on probing rate, IL-2, IL-4, and TNF-α. *Lautropia* primarily negatively correlated with the clinical measures of exhaled carbon monoxide concentration, bleeding on probing rate, cotinine level, and pocket depth.

## DISCUSSION

It is apparent from this study that e-cig use promotes a unique periodontal microbiome, one that contains distinctive features yet shares similarities with those of both conventional cigarette users and nonsmokers. The duration of e-cig use is a strong driver of subgingival microbiome composition over flavoring additions or nicotine concentration, indicating that basal e-cig components exert specific selection pressures on the SGP microbial community (36). Indeed, while this longitudinal study of chronic e-cig users demonstrated an increase in α-diversity with ongoing use, a unique e-cig user microbial community was maintained compared to those of conventional smokers and nonsmokers.

The observed increase over time in α-diversity for all cohorts may indicate a progression in periodontitis due to rare taxa becoming more abundant and thus more likely to be captured in sequencing efforts (22, 23). Accordingly, all cohorts contained individuals that progressed in periodontitis, with several ES patients progressing from a mild to moderate diagnosis. The large representation of initial disease severity and progressed disease states in patients from every cohort provided a rare opportunity to investigate changes in a dysbiotic microbiome due to habit. Xu et al. compared only clinical measures and demographics between these patients and found that the progression of periodontal severity between visits was significantly worse for the CS and ES cohorts than for the NS cohort (7).

Interestingly, all cohorts showed similar magnitudes of increase in α-diversity. Yet notably, significant differences in microbial community structure were observed between all cohorts. This observation may be due to most participants presenting some level of periodontitis and a likely habit-specific dysbiosis at the onset of the study, leaving little room for significant changes in microbial community structure between visits within a cohort. These results demonstrate that although richness increased between visits, habit use impacted microbiome structure differentially and resulted in a unique microbiome for each cohort.

Commonalities existed in the abundances of specific taxa between e-cig users and conventional smokers and e-cig users and nonsmokers. Interestingly, while all cohorts shared about a fifth of total ASVs, the ES cohort shared ∼1.5 times as many ASVs with smokers or nonsmokers as were shared between smokers and nonsmokers. Out of 59 genera above 0.1% relative abundance in at least one cohort, a quarter of them demonstrated significant differences between one cohort and the other two, thus serving as a unique feature of the given cohort (e.g., *Cardiobacterium*, *Veillonella*, Streptococcus, *Fusobacterium*, *Leptotrichia*, and *Neisseria*). These results suggest that habit drives unique abundance patterns, yet e-cig use influences the growth of some microbial taxa in a manner akin to cigarette smoking.

Similarities between smokers and e-cig users included the enrichment of the genera *Selenomonas* and *Leptotrichia* with respect to the NS cohort, with *Leptotrichia* significantly enriched. These genera were also positively correlated with clinical measures and cytokines, clustering together based on correlation patterns with cytokines, particularly IL-1β. *L. wadei* and *L. hongkongensis* clustered with Corynebacterium matruchotii as important features for model accuracy and were generally enriched in the CS and ES cohorts compared to the NS cohort. In contrast, the genus *Corynebacterium* was uniquely enriched in the ES cohort. *L. wadei* and *L. hongkongensis* are associated with caries, gingivitis, periodontitis, and smoking (43–45). *Corynebacterium* taxa, including *C. matruchotii*, are considered a cornerstone of dental biofilm formation, enabling close associations with other organisms (46, 47). Indeed, e-cig aerosols can promote a microenvironment on enamel that is favorable to microbial adhesion and biofilm formation (48). *Selenomonas* species, including Selenomonas sputigena, can also contribute to biofilm formation by coaggregating with multiple species and can be associated with generalized aggressive periodontitis (49–51).

The epibiotic disease-associated *Saccharibacteria* (G-1, G-2, and G-5) were enriched in the CS and ES cohorts with respect to nonsmokers. Additionally, these genera positively correlated with proinflammatory cytokines and clinical measures. There is evidence that taxa closely related to *L. wadei* can serve as hosts for the disease-associated *Saccharibacteria* (52). Interestingly, *P. propionicum* (numerous ASVs found in this study) is also a host for *Saccharibacteria* species (52, 53). Uniquely, *Actinomyces* bacteria, also known hosts for *Saccharibacteria* (52), were most abundant in ES and relatively depleted in CS and NS. Perhaps e-cig use promotes a habit-specific shift in the balance between *Saccharibacteria* and their bacterial hosts in a manner that promotes the enrichment of *Saccharibacteria* and drives dysbiosis. These results suggest that these taxa may play important roles in structuring the periodontal microbiome of e-cig and cigarette users and potentially benefiting from eliciting host inflammatory responses, with e-cig use providing unique opportunities for dysbiosis.

Two uniquely dominant taxa in e-cig users, *Fusobacterium* and *Bacteroidales* (G-2), are anaerobic and known to be associated with periodontitis, with *Bacteroidales* (G-2) more associated than Porphyromonas gingivalis (24, 54, 55). *Fusobacterium* positively correlated with IFN-γ, IL-12p70, and IL-2 and is known to be enriched in periodontitis as an important component of periodontal biofilms (47, 54–57). The significant enrichment of these organisms provides evidence that e-cig use may promote an SGP community enriched in pathogens but in a uniquely dysbiotic manner compared to chronic conventional cigarette use. A previous study looking at the salivary microbiome and e-cig use found that the phylum *Fusobacteria* is significantly depleted in conventional smokers, with similar relative abundances between e-cig users and nonsmokers (6), suggesting that habitual use may have habitat-specific effects.

Further, we used supervised machine learning to examine the uniqueness of the microbiome of each cohort (58–60). Samples from the ES cohort were much less predictable, being incorrectly classified as being from CS or NS over half the time, with a propensity for being classified as from CS. Previous classifiers have had difficulty distinguishing e-cig users, dual users, and former smokers (36), further demonstrating the similarity of the different users’ microbiomes. In support of this, *Lautropia*, *Cardiobacterium*, *Ottowia*, *Anaeroglobus*, *Stomatobaculum*, and *Mitsuokella* all had patterns of relative abundance that suggest that in some ways, the ES microbiome exists as an intermediary state between those of the conventional smoker and nonsmoker. This further suggests that the ES SGP microbiome resembles a mixture of the CS and NS states, with more similarities to the CS state.

IL-4 and IL-1β were significantly decreased in the ES cohort (compared to CS), but TNF-α was uniquely significantly elevated in the ES cohort. IL-4 tends to be reduced in periodontitis and increases after nonsurgical intervention (61), suggesting that taxa present in the dysbiotic periodontal microbiome actively suppress host immune responses (e.g., *Saccharibacteria* discussed above). IFN-γ, IL-1β, and TNF-α are elevated in chronic periodontitis compared to healthy controls (61). In a previous study, IFN-γ and IL-2 were elevated in e-cig users with respect to nonsmokers (62). TNF-α has been shown to be elevated in e-cig users with respect to nonsmokers (63), even when asthmatic smokers temporarily use e-cigs (64). IL-10 can also be elevated by e-cig use (64). A quick proinflammatory response after device use is observed in periodontally healthy e-cig users, one that is on par with the response of patients with severe periodontitis (36). Interestingly, several ASVs classified as *Rothia* or *Actinomyces* were important features for classifying samples: these genera clustered together based on correlation patterns with cytokines, and they were most relatively abundant in the ES cohort, suggesting that chronic e-cig use may uniquely impact these genera. These results suggest a unique host response to e-cig use and/or to an e-cig use-promoted microbial community.

Our results demonstrate that the e-cig user’s subgingival microbiome is a unique amalgamation of microbiota, containing similarities to those of both conventional smokers and nonsmokers. Due to many shared features with the conventional smoker’s microbiome and considering the widespread promotion of e-cigarettes as a “healthier” alternative to or replacement for conventional cigarettes (21, 65–67), our results show that e-cigarette use may promote a healthier SGP microbiome with respect to that of smokers but not compared to that found with never smoking in the first place. The uniqueness of the e-cigarette periodontal microbiome indicates a need for further research into this relatively novel microbial consortium, obtained through the adoption of a newly acquired human habit, and how biotic and abiotic components synergistically impact oral health and disease.

## MATERIALS AND METHODS

### Human subject recruitment and sample collection.

Ethical approval for the study was obtained from the Institutional Review Board of New York University Langone Medical Center. Details of subject enrollment, recruitment, and eligibility criteria were previously published (7) and can be found in Text S1. All subjects underwent clinical assessment for periodontitis at both study visits (6 months apart). A 6-month time frame was chosen to allow for periodontal disease progression (68, 69). Periodontitis state (mild/moderate/severe) was determined based on the definition given by the CDC in collaboration with the American Academy of Periodontology (7, 70). Only subjects with at least mild periodontitis at enrollment were recruited (Text S1). Subgingival plaque (SGP) samples were collected as explained in Text S1 and stored at −80°C until further processing. Cohorts were assigned based on single habit use, duration, frequency, and carbon monoxide levels (Text S1). Nonsmokers stated that they never smoked or used an e-cig in their lifetime and were excluded from the study if found to have ≥7 ppm breath carbon monoxide levels. Clinical measurements were obtained as described by Xu et al. (7) and in Text S1.

### Multiplex immunoassay cytokine measurements.

SGP cytokine and chemokine levels were quantified using the V-Plex human proinflammatory panel 1 kit (10-Plex) from Meso Scale Discovery (MSD; Rockville, MD) according to the manufacturer’s instructions and as described previously (6). Standards, the control pack, and 168 SGP samples from visit 1 and visit 2 were quantified. Samples falling outside the detectable range of the assay were removed from further analysis.

### Microbiome DNA extraction and sequencing.

Genomic DNA from SGP samples was extracted as explained previously using the MoBio Power fecal kit (MoBio Laboratories Inc., Carlsbad, CA) and quantified (6, 71). For 16S rRNA gene library preparation, the V3-V4 region of the 16S rRNA gene was amplified from 10 ng/μL of microbial SGP genomic DNA as described previously (6, 71). Libraries were pooled in equimolar amounts, and pooled amplicon libraries were denatured, diluted, and sequenced on an Illumina MiSeq platform using the MiSeq reagent kit v3 (600 cycles) following the 2 × 300-bp paired-end sequencing protocol. Negative controls were handled exactly as samples and included for all sequencing runs. Negative-control sequence data are deposited with sample data.

### 16S rRNA gene sequence data analysis.

Demultiplexed FASTQs from Illumina were imported into QIIME2 (v2020.2) (72). DADA2 (73) was used to denoise reads and generate the amplicon sequence variant (ASV) table with the following parameters: *--p-trim-left-f 17 --p-trim-left-r 21 --p-trunc-len-f 300 --p-trunc-len-r 196*. A phylogenetic tree was constructed via the *qiime phylogeny align-to-tree-mafft-fasttree* command. A naive Bayes machine learning classifier against the Human Oral Microbiome Database (v15.2) 16S rRNA gene RefSeq database was trained via *q2-feature-classifier* with *fit-classifier-naive-bayes*. Taxonomic assignment was done by using the *classify-sklearn* in *q2-feature-classifier*. Approximately 10% of samples were removed due to low read depth, resulting in samples below 1,001 reads and the patient-matched corresponding-visit sample being culled from further analysis to avoid biases due to sampling depth (74). A rarefied ASV feature table was generated using the *qiime-feature-table rarefy* command with *--p-sampling-depth 1001* and outputted in BIOM format (v2.1) (75). We filtered out 34 samples from 17 subjects who were missing a sample from one of the study visits in the rarefied table, resulting in 168 samples from 84 subjects retained for the downstream analysis, with a total of 3,279 ASVs. The BIOM table, taxonomy file, and phylogenetic tree file were exported for statistical analyses and generation of plots performed in an R environment (v4.0.3), using phyloseq (v1.34.0) (76) and ggplot2 (v3.3.5) (77).

Using the rarefied ASV tables, we utilized the Qiime2 plugin q2-sample-classifier (58, 78) to facilitate supervised machine learning to identify patterns in the relative abundances of ASVs in each cohort and to determine if those patterns were strong enough to predict sample inclusion in a cohort accurately. We trained and tested the classifier using *sample-classifier classify-samples* with the following parameters: --p-n-estimators 1000 --p-estimator RandomForestClassifier; otherwise, the default settings were applied.

### Statistical methods.

Significant differences in cytokine and clinical measures between cohorts were assessed using Kruskal-Wallis H test followed by a *post hoc* Dunn’s test, with multiplicity-adjusted *P* values reported using Prism (v9.0.0). A *P* value of <0.05 was considered significant for all statistical tests performed in this study.

α-Diversity was evaluated using picante (v1.8.2) (79). A paired-sample Wilcoxon signed-rank test was used to compare means to test for significant differences in α-diversity within a cohort over time. To test for significant differences in α-diversity between cohorts, a Mann-Whitney U test was used to compare means.

To test for significant differences in relative abundances of taxa across cohorts, samples from both visits were grouped within a cohort, and pairwise comparisons were made using a Mann-Whitney U test. Linear discriminant analysis effect size (LEfSe) was also used to identify taxa that significantly differed in relative abundance across cohorts (80). Relative-abundance hierarchical heat maps were generated by pheatmap (v1.0.12). For evaluating changes in β-diversity, a principal-coordinate analysis (PCoA) was performed on Bray-Curtis dissimilarity metrics, and one-way permutational multivariate analysis of variance (PERMANOVA) was used to test for significance using the *Vegan* r package (v2.5-7).

Pearson correlation coefficient was calculated to assess the relationships among the relative abundance of the top 20 genera, inflammatory cytokine levels, and clinical measures using the Hmisc r package (v4.5.0). A correlation heat map was generated using the *pheatmap* r package (v1.0.12), and the correlation chord diagram was plotted using the *circlize* r package (v0.4.13). In addition, correlation plots were visually assessed for relationships between clinical measures, cytokine concentration, and taxon relative abundance.

### Data and material availability.

All data needed to evaluate the conclusions in this paper are present in the paper and the supplemental material or are provided elsewhere. All data underlying this study will be made available upon publication. Raw sequence FASTQ files will be made available on the NCBI database under BioProject number PRJNA771337. The BIOM table, taxonomy file, and phylogenetic tree files generated with QIIME2 and scripts used to produce figures are available on GitHub (https://github.com/Fangxi-Xu/E-cigarettes_SGP_Microbiome). Any additional data or material transfer agreements can be provided by Deepak Saxena pending scientific review and a completed material transfer agreement. Requests for the data should be submitted to Deepak Saxena.
