# Supplementary material for: Electronic Cigarette Use Promotes a Unique Periodontal Microbiome
Source: mBio. 2022 Feb 22;13(1):e00075-22. doi: 10.1128/mbio.00075-22 (PMC8903898; doi:10.1128/mbio.00075-22)
Supplement: TEXT S1 [file mbio.00075-22-s0001.docx]

Supplemental text S1.

**Eligibility and classification criteria of the patient population, also detailed in Xu et al., 2021 (1).**

| **Eligibility** | **Criteria** |
| --- | --- |
| Inclusion | Patients were required to be 21 years of age, have a minimum of 16 teeth, including eight posterior teeth, and have mild, moderate, or severe periodontal disease. |
| Exclusion | Patients were excluded from the study if they had:  (a) a medical condition (including uncontrolled diabetes and HIV).  (b) recent febrile illness that delays or precludes participation.  (c) pregnancy or lactation.  (d) history of radiation therapy to the head and neck region.  (e) antibiotic use or professional dental cleaning within 1 month.  (f) enrolled in other studies.  (g) presence of oral mucosal lesions, such as leukoplakia, herpes labialis, and candidiasis. |
| **Periodontitis Status** | **Criteria** |
| Mild | ≥ two interproximal sites with ≥3 mm attachment loss, and ≥2 mm interproximal sites with pocket depth ≥ 4 mm (not on the same tooth), or one interproximal site with pocket depth ≥ 5 mm. |
| Moderate | ≥ two interproximal sites with ≥4 mm attachment loss (not on the same tooth), or ≥2 interproximal sites with PD ≥ 5 mm, also not on the same tooth. |
| Severe | ≥ two interproximal sites with ≥6 mm attachment loss (not on the same tooth), and one or more interproximal site(s) with ≥5 mm pocket depth. |
| **Study Groups** | **Definition** |
| CS | Cigarette smokers: at the time of the study, smoked at least 10 cigarettes daily for a period of 12 months or more, did not use e-cigarettes. |
| ES | Electronic cigarette user: used a minimum of 0.5–1 e-cigarette daily for a minimum of the last 6 months, did not use conventional cigarettes. |
| NS | Nonsmokers*: never smoked a cigarette or used an e-cigarette in their lifetime. |
| Note:  * Nonsmoker patients were excluded from the study if their carbon monoxide level was at or above seven parts per million (ppm). | |

**Study Procedures**

| **Biological Sample Collection** | **Collection procedure** |
| --- | --- |
| subgingival plaque (SGP) | Subgingival plaque samples were collected before periodontal probing by the study clinician using the single stroke technique with sterile Gracey mini-curette from the distal and mesial aspects of eight posterior teeth at baseline and follow-up visits. Plaque samples from disto and mesio locations from a single tooth were pooled to assess the oral microbiome. The samples were placed into individual sterile 2 mL microcentrifuge tubes with transport (TE) buffer and kept on ice until delivered to the lab, where PMFS buffer and aprotinin were added, labeled, and stored at -80°C until further processing. |
| Note: Subgingival plaque samples are the only samples analyzed for 16S rDNA sequencing and cytokine analysis in the manuscript. See Pushalkar et al., 2020 (2) for data derived from saliva samples obtained on visit 1. | |
| **Study Procedures** | **Description** |
| medical and dental history | The medical and dental histories at v1 included a history of current and past conditions, prior surgeries, and current symptoms. Interim medical and dental histories were also evaluated at v2 and included any new medical and dental conditions and/or symptoms since the previous visit. Concomitant medications were also recorded at both visits. Metadata was submitted in RedCap database. If subjects were found to violate the study's eligibility criteria, they were removed. |
| Periodontal exam | The periodontal exam included standard clinical measurements of bleeding on probing (BoP), probing depth (PD), and clinical attachment loss (CAL) and were completed by a calibrated periodontist. |
| Questionnaire | Each patient was asked to complete the CDC periodontal screening questionnaire at every study visit and responses were recorded in RedCap database. |
| Carbon Monoxide levels | Carbon monoxide (CO) levels were tested by CO Smokerlyzer (Smokerlyzer, Covita, Santa Barbara, CA) according to the manufacturer's instructions. Participants were instructed to inhale deeply and hold their breath for fifteen seconds before slowly exhaling into the device. |
| Note: The study procedures listed above took place at both visit 1 and visit 2. | |

Summary:

The extensive clinical protocol and analysis is presented by Xu et al. 2021. The clinical data used in this manuscript are presented in Figure 1 of the main text.

1. Xu F, Aboseria E, Janal MN, Pushalkar S, Bederoff MV, Vasconcelos R, Sapru S, Paul B, Queiroz E, Makwana S, Solarewicz J, Guo Y, Aguallo D, Gomez C, Shelly D, Aphinyanaphongs Y, Gordon T, Corby PM, Kamer AR, Li X, Saxena D. 2021. Comparative Effects of E-Cigarette Aerosol on Periodontium of Periodontitis Patients. Frontiers in Oral Health 2.

2. Pushalkar S, Paul B, Li Q, Yang J, Vasconcelos R, Makwana S, González JM, Shah S, Xie C, Janal MN, Queiroz E, Bederoff M, Leinwand J, Solarewicz J, Xu F, Aboseria E, Guo Y, Aguallo D, Gomez C, Kamer A, Shelley D, Aphinyanaphongs Y, Barber C, Gordon T, Corby P, Li X, Saxena D. 2020. Electronic Cigarette Aerosol Modulates the Oral Microbiome and Increases Risk of Infection. iScience 23:100884.
