# Supplementary figures and images for: Electronic Cigarette Use Promotes a Unique Periodontal Microbiome
Source: mBio. 2022 Feb 22;13(1):e00075-22. doi: 10.1128/mbio.00075-22 (PMC8903898; doi:10.1128/mbio.00075-22)

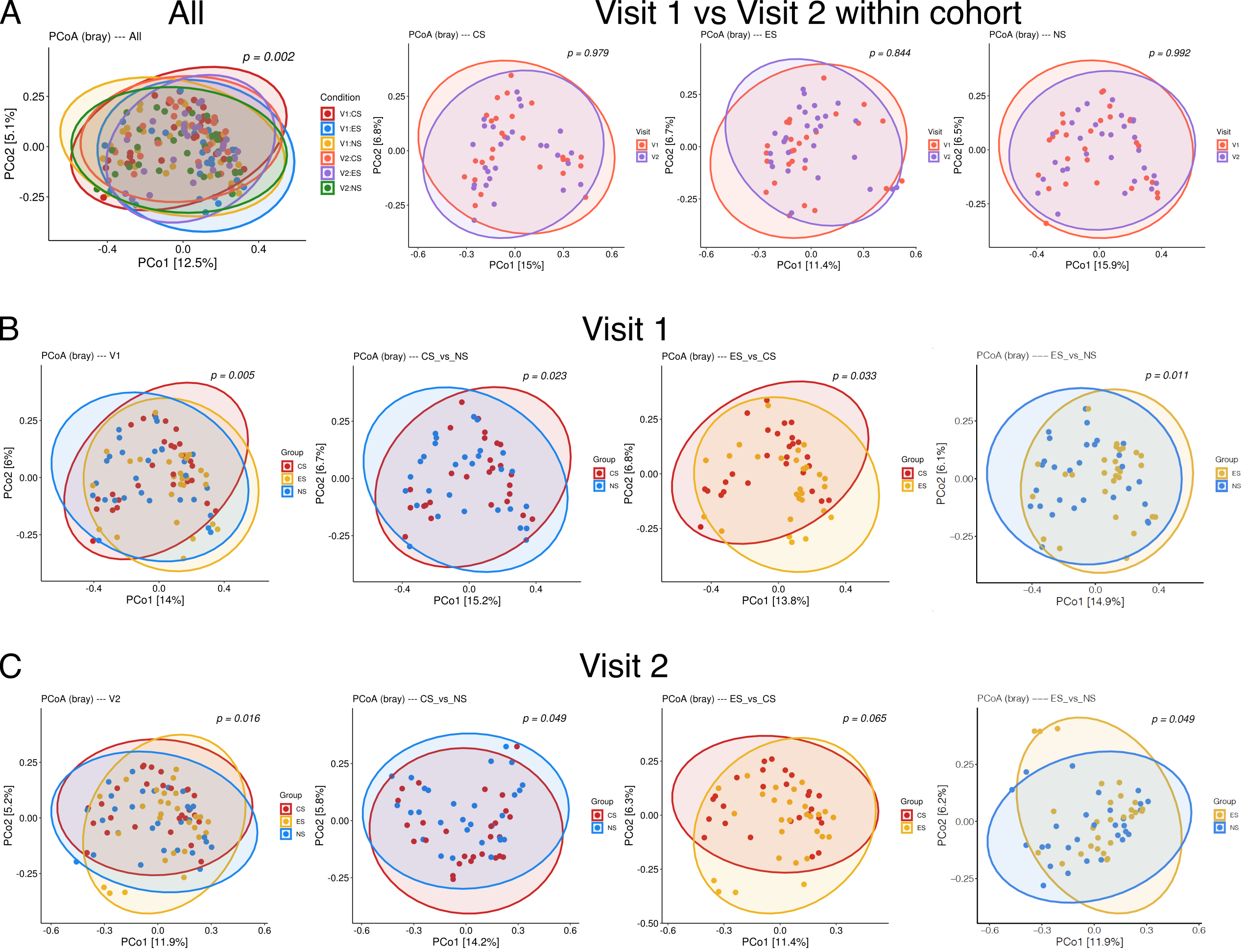

Supplement: FIG S2 [file mbio.00075-22-sf002.tif]
